# The Development of Microbiota and Metabolome in Small Intestine of Sika Deer (*Cervus nippon*) from Birth to Weaning

**DOI:** 10.3389/fmicb.2018.00004

**Published:** 2018-01-23

**Authors:** Zhipeng Li, Xiaoxu Wang, Ting Zhang, Huazhe Si, Weixiao Nan, Chao Xu, Leluo Guan, André-Denis G. Wright, Guangyu Li

**Affiliations:** ^1^Jilin Provincial Key Laboratory for Molecular Biology of Special Economic Animals, Institute of Special Animal and Plant Sciences, Chinese Academy of Agricultural Sciences, Changchun, China; ^2^Department of Agricultural, Food and Nutritional Science, University of Alberta, Edmonton, AB, Canada; ^3^School of Animal and Comparative Biomedical Sciences, University of Arizona, Tucson, AZ, United States

**Keywords:** ruminant, small intestine, microbiota, colonization, metabolome, development

## Abstract

The dense and diverse community of microorganisms inhabiting the gastrointestinal tract of ruminant animals plays critical roles in the metabolism and absorption of nutrients, and gut associated immune function. Understanding microbial colonization in the small intestine of new born ruminants is a vital first step toward manipulating gut function through interventions during early life to produce long-term positive effects on host productivity and health. Yet the knowledge of microbiota colonization and its induced metabolites of small intestine during early life is still limited. In the present study, we examined the microbiota and metabolome in the jejunum and ileum of neonatal sika deer (*Cervus nippon*) from birth to weaning at days 1, 42, and 70. The microbial data showed that diversity and richness were increased with age, but a highly individual variation was observed at day 1. Principal coordinate analysis revealed significant differences in microbial community composition across three time points in the jejunum and ileum. The abundance of *Halomonas* spp., *Lactobacillus* spp., *Escherichia*–*Shigella*, and *Bacteroides* spp. tended to be decreased, while the proportion of *Intestinibacter* spp., *Cellulosilyticum* spp., *Turicibacter* spp., *Clostridium sensu stricto* 1 and *Romboutsia* spp. was significantly increased with age. For metabolome, metabolites separated from each other across the three time points in both jejunum and ileum. Moreover, the amounts of methionine, threonine, and putrescine were increased, while the amounts of myristic acid and pentadecanoic acid were decreased with age, respectively. The present study demonstrated that microbiota colonization and the metabolome becomes more developed in the small intestine with age. This may shed new light on the microbiota-metabolome-immune interaction during development.

## Introduction

Ruminant livestock is an important component in agriculture sector due to the milk, meat, and fiber they produce for human use, but it also increases the consumption of feed resources in order to meet the demands of the growing human population (Eisler et al., 2014). Therefore, improving feed efficiency is critical for developing sustainable ruminant livestock. The growing body of evidence clearly demonstrates that the highly dense and diverse microbial consortium residing in the gastrointestinal tract (GIT), plays fundamental roles in nutrient metabolism (Wu et al., 2016), as well as intestinal physiological development and functions (Bäckhed et al., 2015). Therefore, understanding the GIT microbiota in-depth not only can help to increase feed utilization efficiency, animal health, and production, and but also may provide guidelines to manipulate the GIT fermentation.

Manipulating the GIT fermentation, within the rumen, has been attempted through microbial programming on adult ruminants, with limited or short-term effects (Yanez-Ruiz et al., 2015; Malmuthuge and Guan, 2017a). One main reason is that the GIT microbiota of adult ruminants, displays some resistance to change, as the original composition of the rumen microbiota is usually restored after the manipulation (change in diet, feed additive, etc.) is discontinued (Weimer et al., 2010; Weimer, 2015). In contrast, manipulation of the pre-ruminant GIT microbiota has resulted in persistent and long-term effects (Abecia et al., 2013, 2014). In addition, it has been suggested that the microbial colonization in the GIT, immediately after birth, plays a greater and more lasting effect of the host (Yanez-Ruiz et al., 2015). These results indicate that the early life ruminant provides a unique opportunity for potential manipulation of such a complex microbial ecosystem in the GIT. Therefore, examining colonization of the GIT microbiota is crucial to develop successful microbial programming or methods.

For new born ruminants, most studies focused on the development of rumen function and microbial succession (Li et al., 2012; Rey et al., 2012, 2014; Wu et al., 2012; Jami et al., 2013; Jiao et al., 2015a). Furthermore, the gut microbiota of new born and/or young ruminants has been mainly studied from fecal samples (Uyeno et al., 2010; Oikonomou et al., 2013; Myer et al., 2016). However, since we now know that the microbial composition can significantly vary depending on the region of the GIT, using only fecal samples would not be the best option for examining the microbiota from the small intestine, specifically the jejunum and ileum (Li et al., 2014; Malmuthuge et al., 2014). Of importance, in newborn ruminants, the small intestine serves not only as the main site of liquid feed digestion, but it also plays a role in nutrition absorption and immune development (Ruth and Field, 2013). Thus, it is important to explore the colonization of the microbiota in the small intestine of ruminants. Because only a few studies have used samples from the small intestine (Malmuthuge et al., 2014; Jiao et al., 2016), there is still a lack of the knowledge about the sequential microbial colonization in the small intestine of new born ruminants. In addition to studies describing the gut microbial composition of pre-weaned ruminant, the low-molecular-weight metabolites generated by small intestinal microbiota are absorbed by the intestinal lumen, thereby playing a vital role in ruminant health. Despite some studies investigating the specific metabolites during early life, such as short chain fatty acids and enzymes (Castro et al., 2016a; Jiao et al., 2016), to our knowledge, there are no reports examining the global metabolites (metabolome) produced by microbiota in small intestine.

Sika deer (*Cervus nippon*) is an important species as it produce the velvet antler, the traditional Chinese medicine, which also yields high quality meat and skin. There is approximately 550,000 head of farmed sika deer in China now. In previous studies, we have examined the rumen microbiota of sika deer fed different feeds (Li et al., 2013, 2015a,b). However, very little information is known about the development of the bacterial community in the small intestine of sika deer. Therefore, in the present study, our objectives were to examine the small intestine, including the jejunum and ileum for microbial colonization at days 1, 42, and 70, and to describe the metabolome development in both the jejunum and ileum.

## Materials and Methods

### Animals, Management, and Diets

All animal-specific procedures were approved and authorized by the Chinese Academy of Agricultural Sciences Animal Care and Use Committee, and the Institute of Special Animal and Plant Sciences Wild Animal and Plant Subcommittee (ISAPSWAPS2016000302).

A total of 15 neonatal sika deer (*C. nippon*) were used in this study. The juvenile animals were kept with their dams, which suckled their young until weaning (day 60). During weaning, the juvenile animals also had access to forage. After weaning, the young animals consumed forage and concentrate. All animals had daily access to clean water. On days 1, 42, and 70, after birth, 5 animals were sacrificed. To avoid the mixed contents from the different GIT regions, the animal was keep in a natural position, and the different regions were tied off using cotton rope. The jejunum and ileum contents were collected, and snap frozen in liquid nitrogen prior to storage at -80°C for the further analysis.

### Genomic DNA Extraction, High-Throughput Sequencing and Sequences Analysis

Total microbial genomic DNA was extracted from the jejunum and ileal contents of each animal using the QIAamp DNA Stool Mini Kit (QIAGEN, Valencia, CA, United States) according to the manufacturer’s instructions.

The bacterial primer 341F and 806R, containing a 6-base barcode, the Illumina adapter sequence, and regions for binding of the sequencing primers, were used to amplify the V3–V5 region of the 16S rRNA gene (Human Microbiome Project Consortium, 2012). The target region was applied using a Phusion high fidelity DNA polymerase (NEB, United Kingdom) according to the previous protocol (Human Microbiome Project Consortium, 2012). The resulting amplicons were purified using a QIAquick PCR Purification Kit (QIAGEN, Valencia, CA, United States), and the purified amplicons were quantified using a QuantiFluor^®^-P Fluorometer (Promega, CA, United States), pooled in equimolar concentrations. PhiX Control library (Illumina, 20%) was combined with the amplicon library, and then sequenced on an Illumina PE MiSeq 250 platform to generate paired 250-bp reads.

The read pairs were extracted and concatenated according to the barcodes for each paired read from each sample to generate the contigs. The contigs were trimmed and filtered for quality control using the following criteria: sequences with an average quality < 25 over a 50 bp sliding window were rejected; the minimum quality score was 25; the maximum number of errors in the barcode was 0; the maximum length of homopolymer run was 6; the number of mismatches in the primer was 0; ambiguous and unassigned characters were excluded. The generated sequences were analyzed using QIIME 1.7.0 (Caporaso et al., 2010). In brief, the sequences were clustered into operational taxonomic units (OTUs) using UPARSE at 97% sequence identity (Edgar, 2013). The singletons were removed, and the potential chimera sequences were removed using UCHIME (Edgar et al., 2011). The representative sequences of the OTUs were assigned against the SILVA database (version 123) using the RDP classifier with a 0.80 confidence threshold (Wang et al., 2007; Quast et al., 2013). The phylogenetic tree was constructed using FastTree (Price et al., 2009). We also rarefied the data of each sample to 19,958 sequences without removing singletons (**Supplementary Figure S1**). After that, Chao1, Shannon and Simpson indices, and Good’s coverage were subsequently calculated using QIIME 1.7.0 (Caporaso et al., 2010).

Principal coordinate analysis (PCoA) based on unweighted unifrac distance (an investigation into the presence and absence of bacterial lineages), weighted unifrac distance (which takes relative abundances of bacterial lineages into account), and Bray–Curtis distance were applied to compare the jejunum and ileum community at different time points. AMOVA (analysis of molecular variance) was applied to test whether the microbial communities from jejunum and ileum at three time points have the same centroid, and HOMOVA (homogeneity of molecular variance) was used to test whether the genetic diversity are similar between the microbial communities from the jejunum and ileum at three time points. Canonical correlation analysis (CCA) was also applied to identify the bacteria community difference at each time point from jejunum and ileum using the RAM package (Dufrene and Legendre, 1997). The indicator species analysis selected the most representative features for each cluster or group and split these features into the number of clusters being compared. Kruskal–Wallis analysis was used to test the statistical significance of alpha-diversity indices and to confirm the significance of these indicator species. Significance (*p* < 0.05) was based on the Benjamini–Hochberg corrected *p*-value from the Kruskal–Wallis test (False discovery rate = 0.05).

### Profiling the Metabolites of Jejunum and Ileum and Statistics Analysis

The Gas Chromatography-Time-of-flight Mass Spectrometry (GC-TOFMS) was used to characterize the metabolites in the jejunum and ileum according to previously published methods (Li et al., 2016). The Chroma TOF4.3X software (LECO) and LECO-Fiehn Rtx5 database were used to extract raw peak, to filter data baseline, and to align and identify peak (Kind et al., 2009). Noise was removed using an interquartile range, which were standardized by internal standard normalization methods. A criterion of similarity greater than 300 obtained from the LECO/Fiehn Metabolomics library was selected for the further analysis. The SIMCA-P^+^ 14.0 software package (Umetrics, Umea, Sweden) and Metaboanalyst 3.0 platform (Xia et al., 2015) were used for the pattern recognition multivariate analysis. Principal component analysis (PCA), partial least squares discriminant analysis (PLSDA) and orthogonal partial least-squares discriminant (OPLS-DA) analysis were used to visualize the dissimilarities. The metabolites were plotted according to their importance in differentiating the groups, and each compound was assigned a variable importance in the projection (VIP) value. VIP values that exceeded 1.0 and *p* < 0.05 were used to select the significant metabolites. The significant metabolites identified from the above calculations were used to generate a heatmap using the Metaboanalyst 3.0 platform (Xia et al., 2015).

### Accession Numbers

The sequences in the present study were deposited in the SRA database under accession number SRP116263.

## Results

### Summary of High-Throughput Sequencing and Alpha Diversity

**FIGURE 1:**
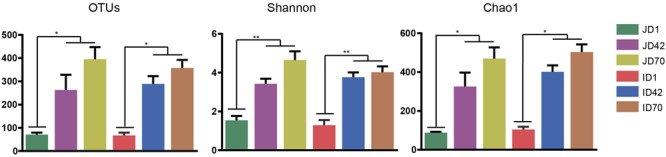
Diversity and richness indices in the jejunum and ileum of neonatal sika deer at days 1, 42, and 70. ^∗^*p* < 0.05, ^∗∗^*p* < 0.01. JD, jejunum day; ID, ileum day.

The present study obtained a total of 856,345 (jejunum = 400,366 and ileum = 455,979) 16S rRNA gene sequences from the jejunum and ileum at three different time points. Sequences ranged from 19,958 to 38,966 sequences for each sample. A total of 1,151 OTUs were identified at 97% sequence identity. The Good’s coverage in the range of 0.992 and 0.999 indicated that more than 99% of the bacterial taxa was captured from the jejunum and ileum. The OTU numbers, Shannon and Chao1 indices in the jejunum and ileum were significantly increased from days 1 to 42 (*p* < 0.05), with an upward trend from days 42 to 70 (**Figure 1**).

### Taxonomic Composition of Jejunum at Days 1, 42, and 70

**FIGURE 2:**
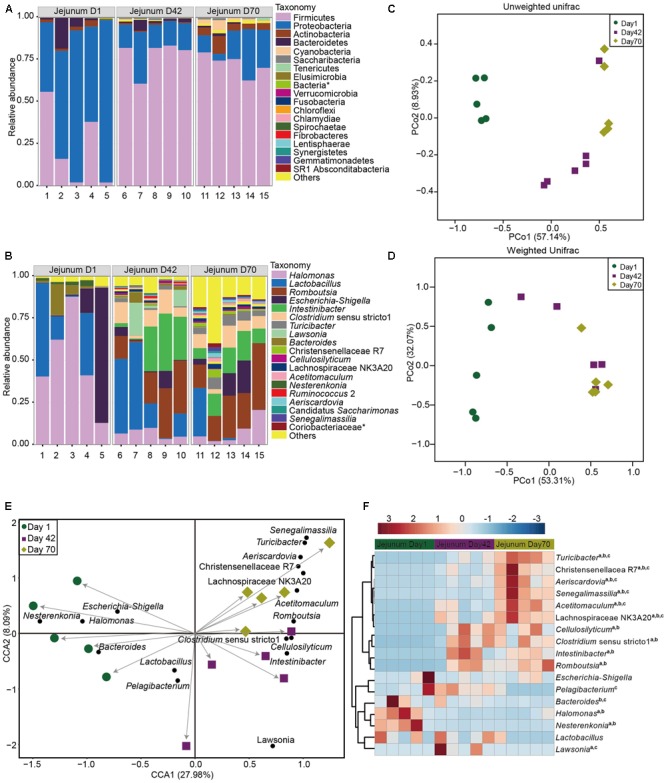
Microbial community composition in the jejunum of sika deer across three time points. Taxonomic composition at the phylum **(A)** and genus levels **(B)**. Principal coordinate analysis (PCoA) of jejunum microbiota based on unweighted unifrac **(C)** and weighted unifrac distance **(D)**. Canonical correlation analysis (CCA) **(E)** and heatmap **(F)** showing the significant taxa of jejunum across three time points. a, b, c indicate the significance between days 1 and 42, between days 42 and 70, and days 42 and 70, respectively. The asterisk means the unclassified bacteria at the family or phylum levels.

A total of 20 phyla were classified based on the identified OTUs (**Figure 2A** and **Supplementary Table S1**). Bacteria belonging to the phyla *Proteobacteria* and *Firmicutes* were predominant, accounting for about 90% of the bacteria at days 1 (69.6 and 22.9%, respectively), 42 (17.7 and 77.6%, respectively) and 70 (17.0 and 72.3%, respectively). At day 1, bacteria belonging to the genus, *Halomonas* (48.9%), was the most dominant, followed by bacteria belonging to the genera *Lactobacillus* (21.4%), *Escherichia*–*Shigella* (19.2%), and *Bacteroides* (5.2%), accounting for about 95% bacterial taxa. At day 42, bacteria belonging to the genus *Lactobacillus* (25.0%) was the predominant genus, followed by the genera: *Romboutsia* (18.8%), *Intestinibacter* (17.8%), *Clostridium sensu stricto* 1 (8.7%), *Halomonas* (6.8%), and *Lawsonia* (6.2%, *Lawsonia intracellularis*, 99% identity) accounting for up to 83.3% of the bacterial community. At day 70, bacteria representing the genus *Romboutsia* (22.9%) was the most abundant genus, followed by the genera *Intestinibacter* (12.2%), *Halomonas* (8.0%), *Clostridium* (7.5%), *Escherichia*–*Shigella* (7.2%), *Lactobacillus* (6.0%), and *Turicibacter* (5.2%), which together made up from 69.0% of the bacterial composition (**Figure 2B**).

Unweighted, weighted unifrac distances and Bray–Curtis distance were applied to examine differences in taxonomic community composition and structure in the jejunum across three time points (**Figure 2** and **Supplementary Figure S2A**). An unweighted unifrac distance analysis showed that the community composition of the jejunum at day 1 was significantly separated from days 42 (AMOVA < 0.01, HOMOVA = 0.25) and 70 (AMOVA < 0.01, HOMOVA = 0.23), explaining 57.14% variation (**Figure 2C**). When a weighted unifrac distance was used, which takes into account the abundance information, the taxonomic composition at day 1 was also distantly separated from the sample of days 42 (AMOVA < 0.01, HOMOVA = 0.081) and 70 (AMOVA < 0.01, HOMOVA = 0.12), with 53.31% variation (**Figure 2D**).

A CCA based on the abundance of an indicator genus, was used to identify influential taxa that facilitated differences across the three time points of jejunum (**Figure 2** and **Supplementary Figure S3**). A total of 17 bacterial taxa were associated with the three different time points (**Figure 2E**). Then we compared the relative abundance of these taxa across three time points. The proportion of *Turicibacter* spp., *Aeriscardovia* spp., *Senegalimassilia* spp., *Acetitomaculum* spp., Lachnospiraceae NK3A20, and Christensenellacea R7 increased linearly from days 1 to 70 (*p* < 0.05, **Figure 2F**). The abundance of *Cellulosilyticum* spp., *Clostridium sensu stricto* 1, *Intestinibacter* spp., and *Romboutsia* spp. were significantly higher at days 42 and 70 than at day 1 (*p* < 0.05), while the abundance of *Halomonas* spp. and *Nesterenkonia* spp. were significantly decreased at days 42 and 70 than at day 1 (*p* < 0.05). Moreover, the abundance of *Escherichia*–*Shigella* and *Lactobacillus* spp. at days 42 (*p* = 0.64 and *p* = 0.36, respectively) and 70 (*p* = 0.26 and *p* = 0.49, respectively) were not significantly different than day 1.

### Microbial Composition of Ileum at Days 1, 42, and 70

**FIGURE 3:**
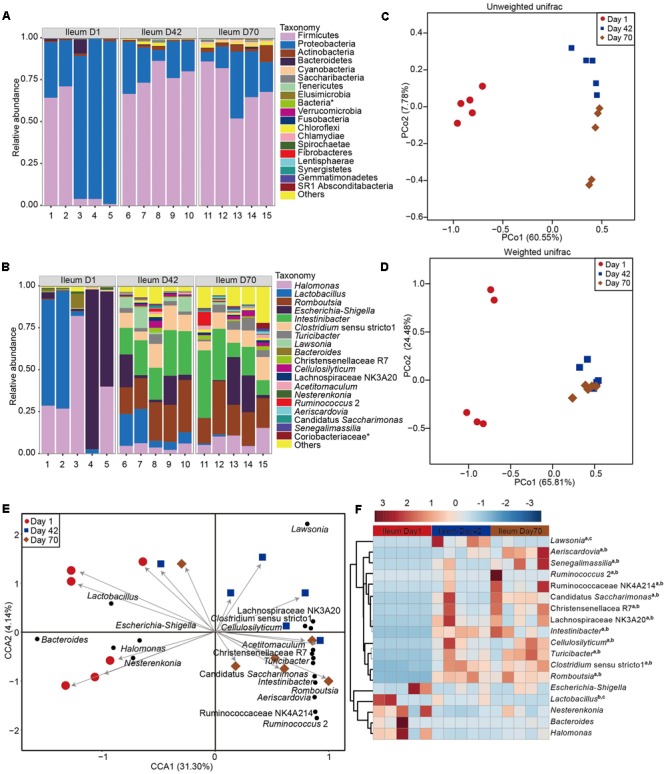
Microbial community composition in ileum of sika deer across three time points. Taxonomic composition at phylum **(A)** and genus level **(B)**. Principal coordinate analysis (PCoA) of ileum microbiota based on unweighted unifrac **(C)** and weighted unifrac distance **(D)**. CCA **(E)** and heatmap **(F)** showing the significant taxa of ileum across three time points. a, b, c indicate the significance between days 1 and 42, between days 42 and 70, and days 42 and 70, respectively. The asterisk means the unclassified bacteria at the family or phylum levels.

A total of 18 bacterial phyla were identified from the ileum (**Figure 3A** and **Supplementary Table S1**). Bacteria belonging to the phyla *Proteobacteria* and *Firmicutes* were the predominant microorganisms, accounting for more than 91% of all taxa at days 1 (67.7 and 29.1%, respectively), 42 (19.7 and 76.7%, respectively) and 70 (20.7 and 70.7%, respectively). At genus level (**Figure 3B**), bacteria from the genus *Halomonas* (35.7%) were the most prevalent, followed by the genera *Escherichia*–*Shigella* (31.4%) and *Lactobacillus* (27.7%), accounting for up to 95% taxonomic composition. At day 42, bacteria representing the genus *Romboutsia* (22.1%) was the most prevalent, followed by the genera *Intestinibacter* (21.8%), *Lactobacillus* (10.9%), *Clostridium sensu stricto* 1 (10.5%), *Escherichia*–*Shigella* (8.7%), *Lawsonia* (6.0%, *Lawsonia intracellularis*, 99% identity) and *Halomonas* (4.7%), accounting for 85% of the taxonomic community. At day 70, bacteria belonging to the genus *Intestinibacter*. (21.1%) was the most prevalent, followed by the genera *Romboutsia* (20.6%), *Escherichia*–*Shigella* (10.6%), *Halomonas* (9.4%), *Clostridium sensu stricto* 1 (9.3%), and *Turicibacter* (4.4%), which together made up from 75% of the taxonomic composition.

The PCoA analysis revealed that the microbial communities from ileum at three time points are varied (**Figure 3** and **Supplementary Figure S2B**). The unweighted unifrac distance analysis showed that the community composition of the ileum at day 1 was distantly separated from that of days 42 (AMOVA < 0.01, HOMOVA = 0.09) and 70 (AMOVA < 0.01, HOMOVA = 0.18), explaining 60.55% variation (**Figure 3C**). A weighted unifrac distance showed that the taxonomic composition between days 1 and 42 (AMOVA < 0.01, HOMOVA = 0.15) or 70 (AMOVA < 0.01, HOMOVA = 0.10) was more pronounced explaining 65.81% variation (**Figure 3D**).

Differences in the taxonomic composition across three time points in ileum of sika deer was identified based on CCA and Lefse analysis (**Figure 3** and **Supplementary Figure S4**). The result of CCA showed that a total of 18 bacterial taxa were associated with the three different time points in the ileum (**Figure 3E**). The abundance of the genera *Escherichia*–*Shigella, Lactobacillus*, and *Halomonas* at day 1 were not significantly different compared to days 42 and 70 (*p* > 0.05). The abundance of bacteria belonging to the bacterial taxa *Aeriscardovia* spp., *Ruminococcus* 2, Ruminococcaceae NK4A2, Candidatus *Saccharimonas* spp., *Christensenellacea* R7, Lachnospiraceae NK3A20, *Intestinibacter* spp., *Cellulosilyticum* spp., *Turicibacter* spp., *Clostridium sensu stricto* 1, and *Romboutsia* spp. at days 42 or 70 were significantly increased compared to day 1 (*p* < 0.05).

### Metabolites in Jejunum and Ileum at Days 1, 42, and 70

**FIGURE 4:**
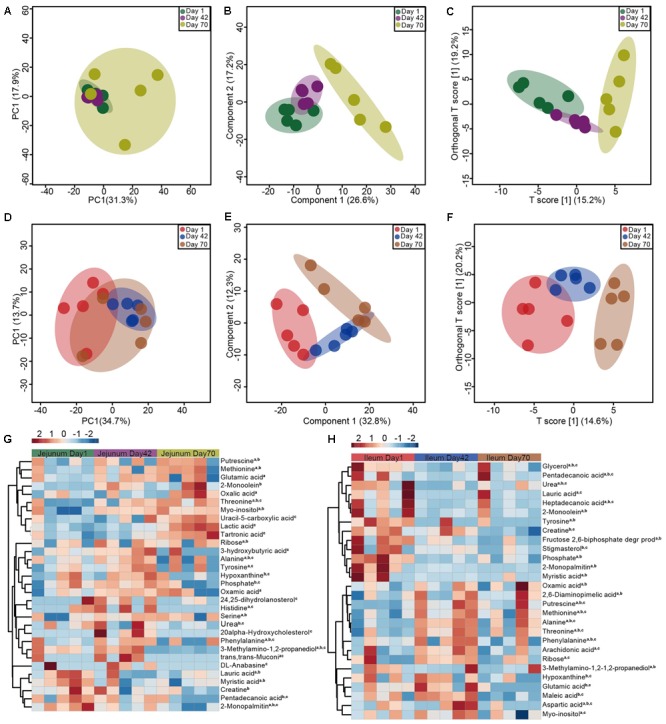
The jejunum and ileum metabolome of sika deer at days 1, 42, and 70. Principal component analysis (PCA), partial least squares discriminant analysis (PLSDA) and orthogonal partial least-squares discriminant (OPLS-DA) analysis revealing the metabolites variation in jejunum **(A–C)** and ileum **(D–F)**, respectively. Heat-map showing the significantly increased or decreased metabolites in jejunum **(G)** and ileum **(H)**, respectively. a, b, c indicate the significance between days 1 and 42, between days 42 and 70, and days 42 and 70, respectively.

A total of 138 reliable compounds were identified from the jejunum and ileum. PCA showed that the metabolites tended to cluster together across the three time points for both jejunum (**Figure 4A**) and ileum (**Figure 4D**), explaining 49.2 and 48.4% variation, respectively. PLSDA and OPLS-DA analyses showed that the metabolites were different across the three time points for jejunum (**Figures 4B,C**) and ileum (**Figures 4E,F**), especially at day 70.

The VIP value exceeded 1.0 is used to identify the significant metabolites. For the jejunum, the amount of threonine was significantly increased from days 1 to 70 (**Figure 4G**). The concentration of lactic acid, methionine, myo-inositol, putrescine and serine was not significantly different from days 1 to 70. While, the amounts of 2-monopalmitin, creatine, myristic acid, and pentadecanoic acid linearly decreased from days 1 to 70. The amounts of alanine, histidine, hypoxanthine, oxamic acid, phenylalanine and tyrosine were increased from days 1 to 42, but were decreased from days 42 to 70.

For metabolites in the ileum (**Figure 4H**), the amounts of glycerol, pentadecanoic acid, urea, lauric acid, heptadecanoic acid, 2-monoolein, phosphate, and myristic acid at day 1 were greater than that at days 42 and/or 72. The amounts of creatine, fructose 2,6-biphosphate, 2-monopalmitin, 3-methylamino-1,2-propanediol, stigmasterol, and tyrosine linearly decreased from days 1 to 70. In contrast, the amounts of oxamic acid, 2,6-diaminopimelic acid, putrescine, methionine, alanine, threonine, phenylalanine, arachidonic acid, and ribose at was lower at day 1 than at days 42 and/or 70.

## Discussion

In the present study, microbial diversity at day 1 was highly variable compared to other days in both the jejunum and ileum (**Figures 1, 2**). This is consistent with previous reports in rumen, ileum, and feces of pre-weaned cattle and goat (Uyeno et al., 2010; Edrington et al., 2012; Li et al., 2012; Jami et al., 2013; Oikonomou et al., 2013; Klein-Jöbstl et al., 2014; Jiao et al., 2016; Dill-McFarland et al., 2017; Malmuthuge and Guan, 2017b). These results indicate that the first colonization events in small intestine may be influenced by many factors including dam’s milk, vaginal birth, salivary microorganisms, or environmental microbiota (Mueller et al., 2015; Yanez-Ruiz et al., 2015; Stephens et al., 2016). However, given that the diet is fully based on dam’s milk after birth, and the newborn sika deer can be considered a non-ruminant from a functional point of view, reflex closure of the reticular (or esophageal) groove forms a passage between the esophagus and omasum to ensure the passage of milk components directly to the small intestine (Drackley, 2008). This suggests a greater role of milk microbiota in early colonization of the jejunum and ileum microbiota. This is consistent with recent correlations in the breast milk bacterial community and infant gut microbiota, that showed infants who received 75% of their total daily milk intake as breast milk, received about 27.7% of the bacteria from breast milk during the first 30 days of life (Collado et al., 2007).

The microbial diversity, richness, and the microbial community structure at days 42 and 70 were significantly different from at day 1, but bacterial diversity was not significant between days 42 and 70 for both the jejunum and the ileum (**Figures 1, 2** and **Supplementary Figures S2, S5**). In accordance with findings from the ileum microbiota of goats (Jiao et al., 2016), age plays a much more important role in affecting the small intestine microbiota, and the succession of a more similar microbial community in older animals may be established before animals are weaned, which may provide new insights to manipulate the gut health during the early colonization period.

The phyla *Proteobacteria* and *Firmicutes* are dominating in the jejunum and ileum over the development of these juvenile sika deer. This agrees with the results from the ileum juvenile goats (Jiao et al., 2016), and disagrees with the results from the small intestine of 3 week old calves (Malmuthuge et al., 2014). However, the proportion of bacteria representing the phylum *Proteobacteria* in jejunum and ileum at day 1 (>65%) is much greater than the results from the ileum of goats fed milk at days 0 and 7 (23.9 and 5.4%, respectively) (Jiao et al., 2016). These results imply that milk is an important factor affecting small intestine microbial composition.

The sequences from *Halomonas* spp., *Lactobacillus* spp., *Escherichia*–*Shigella*, and *Bacteroides* spp. are the dominant bacteria at day 1 that tend to decrease with age (**Figures 2E,F, 3E,F**). *Lactobacillus* spp., *Escherichia*–*Shigella*, and *Bacteroides* spp. bacteria are also abundant in the rumen, jejunum, ileum, colon and feces of calf and goat after birth (Uyeno et al., 2010; Edrington et al., 2012; Oikonomou et al., 2013; Klein-Jöbstl et al., 2014; Castro et al., 2016a,b; Myer et al., 2016). These similarities may be related to the vaginal birth and contact with the vaginal microbiota and, or, influence of the milk bacterial community during feeding (Collado et al., 2007; Santos et al., 2011; Klein-Jöbstl et al., 2014). The common distribution of these microbiota from different ruminants indicates their general importance in the early growth period. For instance, *Lactobacillus* spp. and *Escherichia* spp. are facultative anaerobic bacteria, which can create the anaerobic conditions that allow for the succession and establishment of obligate anaerobes in gut (Kalita et al., 2014; Malmuthuge et al., 2015).

Notably, the sequences from *Halomonas* spp. is highly abundant in the jejunum and ileum after birth (**Figures 2, 3**), which has not been demonstrated in the gut of new born calf and goat (Jiao et al., 2016; Malmuthuge and Guan, 2017b). We also found *Halomonas* spp. was comparatively abundant in milk of both water deer and reindeer (Li et al., 2017). These results indicate that *Halomonas* spp. in the jejunum and ileum of sika deer at day 1, may have been transferred from the sika deer milk, reinforcing the idea that the early microbiota in the small intestine is related to the milk microbiota and the environment. Moreover, *Halomonas* spp. has been demonstrated to inhibit enteric LPS-induced human monocyte activation producing several pro-inflammatory cytokines (Ialenti et al., 2006). It is speculated that *Halomonas* spp. may play a role in facilitating the gut immune development. However, this hypothesis needs to be documented in future study through *in vitro* cultivation approach.

The prevalence of the sequences from *Intestinibacter* spp., *Cellulosilyticum* spp., *Turicibacter* spp., *Clostridium sensu stricto* 1, and *Romboutsia* spp. is significantly increased and dominated in both the jejunum and ileum of sika deer at days 42 and 70 (**Figures 2E,F, 3E,F**). Jiao et al. (2016) found that the bacteria from the genera *Prevotella, Butyrivibrio, Ruminococcus, Fibrobacter*, and SMB53 within the Clostridiaceae family surged in abundance after day 20 in the goat ileum (Jiao et al., 2016). The most prevalent bacterial population in the colon and feces of cattle from weeks 1 to 6 were represented by the genera *Bacteroides* and *Prevotella* (Edrington et al., 2012; Li et al., 2012; Jami et al., 2013; Malmuthuge et al., 2014). While, a higher abundance of bacterial representing the *Bacteroides*–*Prevotella* and *Clostridium coccoides*–*Eubacterium rectale* groups was observed in the feces of dairy calves (Uyeno et al., 2010). These difference may be caused by the different forages used in these studies.

The mucosal immune system is also crucial for the development and rapid colonization of commensal bacteria in the gut (El Aidy et al., 2012; Liang et al., 2016), such as the first physical mucus barrier, containing major mucins, and a glycoprotein secreted from a goblet cell, that also constitutes a carbon and energy source for gut microbiota (Li et al., 2012). For instance, the members of the *Clostridium sensu stricto* 1 genus can consume mucus-derived saccharides as energy sources, such as glucose, to acetate, butyrate, lactate, ethanol, H_2_ and CO_2_ (Bauchart-Thevret et al., 2009). Thereby causing the host to respond with increased production, subsequently thickening the inner mucus layer (Wlodarska et al., 2015). Although little is known about the role of *Intestinibacter* spp. in the gut ecosystem, analysis of the SEED and gut microbial modules functional annotations show *Intestinibacter* spp. is involved in mucin consumption through the degradation of fucose (Forslund et al., 2015). In addition, *Cellulosilyticum* spp. is positively correlated with goblet cell number per μm villus height promoting mucus secretion (McCormack et al., 2017). On the other hand, *Akkermansia muciniphila*, has been described to utilize mucin (Derrien et al., 2004), and is common member of the human gut microbiota with early colonization in human infant (Collado et al., 2007). Thus it is speculated that these bacteria may play a role in the mucin degradation, and are related to normal mucosa development and function.

Interestingly, there is also evidence that these bacterial populations are related on host immune cells for survival. *Turicibacter* spp. is shown to be related to the innate immune and B and T cell populations (Smedman et al., 1999), iNK T cell and marginal zone B cell abundance (Presley et al., 2010), Toll-like receptor-2 (Derrien et al., 2004). *Romboutsia* spp. is suggested to be associated with less severe immune responses as demonstrated by decreasing plasma levels of proinflammatory cytokines (Liang et al., 2016). Additionally, Piccolo et al. (2017) also demonstrated that the *Lactobacillus* spp. in the small intestine help neonatal pigs to develop the gut-associated lymphoid tissue and immune response. Together, these findings indicated that there is a close interaction between the bacterial population and host immune regulation, which is an important factor to influence the succession of the small intestinal microbiota. The limitation of the present study is that the microbiota and transcriptome of small intestine epithelium was not examined, which will provide more accurate and direct evidence to the host immune and microbiota interaction in future studies.

The metabolome results show that the metabolites separated from each other across the three time points for both the jejunum and ileum (**Figure 4**), suggesting that the metabolic capacity of the small intestine also displays age-dependence. This finding is consistent with the observed pattern of the goat ileum enzyme showing that the highest amylase activity is observed at day 42, while xylanase activity increases quadratically from days 28 to 70 (Jiao et al., 2016). However, this is contrast to the previous findings in the rumen. For example, Li et al. (2012) demonstrated that the rumen microbial communities of pre-ruminant calves maintained a stable community function and metabolic potentials while their phylogenetic composition displayed a great tendency for fluctuation (Li et al., 2012). Further, the main fermentative and enzymatic activities are stabilized at 1 month (Rey et al., 2012; Jiao et al., 2015b). These difference may be attributed to the functional heterogeneity of the GIT (Godoy-Vitorino et al., 2012), as the rumen is the main site of diet fermentation, and the small intestine plays an important role in nutrient absorption and maintaining immune homeostasis.

The increased amounts of methionine, threonine, and putrescine, and the decreased amounts of myristic acid and pentadecanoic acid in the jejunum and ileum (**Figure 4**), indicated an increase of food source, as pentadecanoic acid is a marker for intake of milk fat in mouse (Smedman et al., 1999). Moreover, putrescine is a product of bacteria in the gut, which is also mainly dependent on gut microbiota, such as *Clostridia* spp. (Yeruva et al., 2016). Therefore, the increased amount of putrescine suggested that the metabolic activity of microbiota in small intestine of sika deer may be changed. Threonine is a key amino acid in mucin synthesis, accounting for 28–35% of the total amino acids of mucin (Law et al., 2007), and represents 7–11% of the total amino acids in IgA (Sandberg et al., 2007). Additionally, putrescine also plays important role in the maturation and maintenance of the intestinal mucosal barrier and anti-inflammatory actions (Eisler et al., 2014). The increased amount of threonine could also promote mucin synthesis (Faure et al., 2006). These findings reinforce the suggestion that the small intestinal microbiota and metabolome are closely interacted, resulting in gut function and immune development. Further studies are required to identify the derived source of these amino acids, either from microbiota or diet metabolism, and to examine their effects on gut function and immune development *in vivo*.

## Conclusion

In summary, the present study revealed the microbial colonization and metabolome development in the jejunum and ileum of juvenile sika deer. Our results showed that the microbial diversity in both the jejunum and ileum increased with age, implying that age is a key factor in microbial succession. The microbial community composition of juvenile sika deer at day 1 was significantly different than days 42 and 70, indicating the potential role and influence of the dam’s milk microbiota on the colonization of microorganisms in the jejunum and ileum. The increased microbiota and dominant bacteria at days 42 and 70, may be closely associated with immune development of the small intestine. Moreover, the metabolome analysis in the jejunum and ileum also displayed an age-dependent pattern. The varied metabolites across the three time points revealed that the metabolic activity of protein and fat changed with development, which also may be related to the immune development of small intestine. Furthermore, the present study identified significant differences in the bacteria and metabolites in the jejunum and ileum during early life. Moreover, future research should investigate how these metabolites and microbial taxa affect small intestinal function.

## Author Contributions

ZL, XW, TZ, HS, WN, and CX collected the samples. ZL and TZ prepared the samples for analysis. ZL analyzed the data. ZL, A-DGW, LG, and GL wrote and reviewed the manuscript. All authors approved the final manuscript as submitted.

## Conflict of Interest Statement

The authors declare that the research was conducted in the absence of any commercial or financial relationships that could be construed as a potential conflict of interest.

## Supplementary Material

FIGURE S1 Rarefaction curves of the jejunum and ileum microbial community based on 16S rRNA gene sequencing based on a 97% sequence similarity. OTU, operational taxonomic unit.

FIGURE S2 Principal coordinate analysis (PCoA) of jejunum **(A)** and ileum **(B)** microbiota based on Bray–Curtis distance.

FIGURE S3 Taxonomic representation showing the statistically and biologically difference in jejunum across three time points. Differences are represented by the color of the most abundant class (red indicating the samples at day 1, green indicating the samples at day 42, and blue indicating the samples at day 70). The diameter of each circle’s diameter is proportional to the taxa abundance.

FIGURE S4 Taxonomic representation showing the statistically and biologically difference in ileum across three time points. Differences are represented by the color of the most abundant class (red indicating the samples at day 1, green indicating the samples at day 42, and blue indicating the samples at day 70). The diameter of each circle’s diameter is proportional to the taxa abundance.

FIGURE S5 Comparison of the microbiota in jejunum and ileum across three time points using PCoA. *R*^2^- and *p*-values are the results of the multivariate ANOVA analysis based on the Bray–Curtis distance. D, day.

TABLE S1 Species composition in jejunum and ileum across three time points.

## References

[B1] Abecia L. Martín-García A. I. Martínez G. Newbold C. J. Yáñiez-Ruiz D. R. (2013). Nutritional intervention in early life to manipulate rumen microbial colonization and methane output by kid goats postweaning. *J. Anim. Sci.* 91 4832–4840. 10.2527/jas.2012-6142 23965388

[B2] Abecia L. Waddams K. E. Martínez-Fernandez G. Martín-García A. I. Ramos-Morales E. Newbold C. J. (2014). An antimethanogenic nutritional intervention in early life of ruminants modifies ruminal colonization by Archaea. *Archaea* 2014:841463. 10.1155/2014/841463 24803846 PMC3997891

[B3] Bäckhed F. Roswall J. Peng Y. Feng Q. Jia H. Kovatcheva-Datchary P. (2015). Dynamics and stabilization of the human gut microbiome during the first year of life. *Cell Host Microbe* 17 690–703. 10.1016/j.chom.2015.04.004 25974306

[B4] Bauchart-Thevret C. Stoll B. Chacko S. Burrin D. G. (2009). Sulfur amino acid deficiency upregulates intestinal methionine cycle activity and suppresses epithelial growth in neonatal pigs. *Am. J. Physiol. Endocrinol. Metab.* 296 E1239–E1250. 10.1152/ajpendo.91021.2008 19293331 PMC2692405

[B5] Caporaso J. G. Kuczynski J. Stombaugh J. Bittinger K. Bushman F. D. Costello E. K. (2010). QIIME allows analysis of high-throughput community sequencing data. *Nat. Methods* 7 335–336. 10.1038/Nmeth.F.303 20383131 PMC3156573

[B6] Castro J. J. Gomez A. White B. Loften J. R. Drackley J. K. (2016a). Changes in the intestinal bacterial community, short-chain fatty acid profile, and intestinal development of preweaned Holstein calves. 2. Effects of gastrointestinal site and age. *J. Dairy Sci.* 99 9703–9715. 10.3168/jds.2016-11007 27720148

[B7] Castro J. J. Gomez A. White B. A. Mangian H. J. Loften J. R. Drackley J. K. (2016b). Changes in the intestinal bacterial community, short-chain fatty acid profile, and intestinal development of preweaned Holstein calves. 1. Effects of prebiotic supplementation depend on site and age. *J. Dairy Sci.* 99 9682–9702. 10.3168/jds.2016-11006 27720150

[B8] Collado M. C. Derrien M. Isolauri E. de Vos W. M. Salminen S. (2007). Intestinal integrity and *Akkermansia muciniphila*, a mucin-degrading member of the intestinal microbiota present in infants, adults, and the elderly. *Appl. Environ. Microbiol.* 73 7767–7770. 10.1128/AEM.01477-07 17933936 PMC2168041

[B9] Derrien M. Vaughan E. E. Plugge C. M. de Vos W. M. (2004). *Akkermansia muciniphila* gen. nov., sp. nov., a human intestinal mucin-degrading bacterium. *Int. J. Syst. Evol. Microbiol.* 54 1469–1476. 10.1099/ijs.0.02873-0 15388697

[B10] Dill-McFarland K. A. Breaker J. D. Suen G. (2017). Microbial succession in the gastrointestinal tract of dairy cows from 2 weeks to first lactation. *Sci. Rep.* 7:40864. 10.1038/srep40864 28098248 PMC5241668

[B11] Drackley J. K. (2008). Calf nutrition from birth to breeding. *Vet. Clin. North Am. Food Anim. Pract.* 24 55–86. 10.1016/j.cvfa.2008.01.001 18299032

[B12] Dufrene M. Legendre P. (1997). Species assemblages and indicator species: the need for a flexible asymmetrical approach. *Ecol. Monogr.* 67 345–366. 10.2307/2963459

[B13] Edgar R. C. (2013). UPARSE: highly accurate OTU sequences from microbial amplicon reads. *Nat. Methods* 10 996–998. 10.1038/nmeth.2604 23955772

[B14] Edgar R. C. Haas B. J. Clemente J. C. Quince C. Knight R. (2011). UCHIME improves sensitivity and speed of chimera detection. *Bioinformatics* 27 2194–2200. 10.1093/bioinformatics/btr381 21700674 PMC3150044

[B15] Edrington T. S. Dowd S. E. Farrow R. F. Hagevoort G. R. Callaway T. R. Anderson R. C. (2012). Development of colonic microflora as assessed by pyrosequencing in dairy calves fed waste milk. *J. Dairy Sci.* 95 4519–4525. 10.3168/jds.2011-5119 22818466

[B16] Eisler M. C. Lee M. R. Tarlton J. F. Martin G. B. Beddington J. Dungait J. A. (2014). Agriculture: steps to sustainable livestock. *Nature* 507 32–34. 10.1038/507032a 24605375

[B17] El Aidy S. van Baarlen P. Derrien M. Lindenbergh-Kortleve D. J. Hooiveld G. Levenez F. (2012). Temporal and spatial interplay of microbiota and intestinal mucosa drive establishment of immune homeostasis in conventionalized mice. *Mucosal Immunol.* 5 567–579. 10.1038/mi.2012.32 22617837

[B18] Faure M. Mettraux C. Moennoz D. Godin J.-P. Vuichoud J. Rochat F. (2006). Specific amino acids increase mucin synthesis and microbiota in dextran sulfate sodium-treated rats. *J. Nutr.* 136 1558–1564. 10.1093/jn/136.6.1558 16702321

[B19] Forslund K. Hildebrand F. Nielsen T. Falony G. Le Chatelier E. Sunagawa S. (2015). Disentangling type 2 diabetes and metformin treatment signatures in the human gut microbiota. *Nature* 528 262–266. 10.1038/nature15766 26633628 PMC4681099

[B20] Godoy-Vitorino F. Goldfarb K. C. Karaoz U. Leal S. Garcia-Amado M. A. Hugenholtz P. (2012). Comparative analyses of foregut and hindgut bacterial communities in hoatzins and cows. *ISME J.* 6 531–541. 10.1038/ismej.2011.131 21938024 PMC3280141

[B21] Human Microbiome Project Consortium (2012). Structure, function and diversity of the healthy human microbiome. *Nature* 486 207–214. 10.1038/nature11234 22699609 PMC3564958

[B22] Ialenti A. Di Meglio P. Grassia G. Maffia P. Di Rosa M. Lanzetta R. (2006). A novel lipid a from *Halomonas magadiensis* inhibits enteric LPS-induced human monocyte activation. *Eur. J. Immunol.* 36 354–360. 10.1002/eji.200535305 16365914

[B23] Jami E. Israel A. Kotser A. Mizrahi I. (2013). Exploring the bovine rumen bacterial community from birth to adulthood. *ISME J.* 7 1069–1079. 10.1038/Ismej.2013.2 23426008 PMC3660679

[B24] Jiao J. Huang J. Zhou C. Tan Z. (2015a). Taxonomic identification of ruminal epithelial bacterial diversity during rumen development in goats. *Appl. Environ. Microbiol.* 81 3502–3509. 10.1128/aem.00203-15 25769827 PMC4407235

[B25] Jiao J. Li X. Beauchemin K. A. Tan Z. Tang S. Zhou C. (2015b). Rumen development process in goats as affected by supplemental feeding v. grazing: age-related anatomic development, functional achievement and microbial colonisation. *Br. J. Nutr.* 113 888–900. 10.1017/S0007114514004413 25716279

[B26] Jiao J. Wu J. Zhou C. Tang S. Wang M. Tan Z. (2016). Composition of ileal bacterial community in grazing goats varies across non-rumination, transition and rumination stages of life. *Front. Microbiol.* 7:1364. 10.3389/fmicb.2016.01364 27656165 PMC5011132

[B27] Kalita A. Hu J. Torres A. G. (2014). Recent advances in adherence and invasion of pathogenic *Escherichia coli*. *Curr. Opin. Infect. Dis.* 27 459–464. 10.1097/qco.0000000000000092 25023740 PMC4169667

[B28] Kind T. Wohlgemuth G. Lee D. Y. Lu Y. Palazoglu M. Shahbaz S. (2009). FiehnLib: mass spectral and retention index libraries for metabolomics based on quadrupole and time-of-flight gas chromatography/mass spectrometry. *Anal. Chem.* 81 10038–10048. 10.1021/ac9019522 19928838 PMC2805091

[B29] Klein-Jöbstl D. Schornsteiner E. Mann E. Wagner M. Drillich M. Schmitz-Esser S. (2014). Pyrosequencing reveals diverse fecal microbiota in simmental calves during early development. *Front. Microbiol.* 5:622. 10.3389/fmicb.2014.00622 25452753 PMC4233928

[B30] Law G. K. Bertolo R. F. Adjiri-Awere A. Pencharz P. B. Ball R. O. (2007). Adequate oral threonine is critical for mucin production and gut function in neonatal piglets. *Am. J. Physiol. Gastrointest. Liver Physiol.* 292 G1293–G1301. 10.1152/ajpgi.00221.2006 17234895

[B31] Li R. W. Connor E. E. Li C. J. Baldwin R. L. Sparks M. E. (2012). Characterization of the rumen microbiota of pre-ruminant calves using metagenomic tools. *Environ. Microbiol.* 14 129–139. 10.1111/j.1462-2920.2011.02543.x 21906219

[B32] Li Z. Wright A. D. Liu H. Bao K. Zhang T. Wang K. (2015a). Bacterial community composition and fermentation patterns in the rumen of sika deer (*Cervus nippon*) fed three different diets. *Microb. Ecol.* 69 307–318. 10.1007/s00248-014-0497-z 25252928

[B33] Li Z. Wright A. D. G. Liu H. Fan Z. Yang F. Zhang Z. (2015b). Response of the rumen microbiota of sika deer (*Cervus nippon*) fed different concentrations of tannin rich plants. *PLOS ONE* 10:e0123481. 10.1371/journal.pone.0123481 25955033 PMC4425498

[B34] Li Z. Wright A. D. G. Si H. Wang X. Qian W. Zhang Z. (2016). Changes in the rumen microbiome and metabolites reveal the effect of host genetics on hybrid crosses. *Environ. Microbiol. Rep.* 8 1016–1023. 10.1111/1758-2229.12482 27717170

[B35] Li Z. Wright A. D. G. Yang Y. Si H. Li G. (2017). Unique bacteria community composition and co-occurrence in the milk of different ruminants. *Sci. Rep.* 7:40950. 10.1038/srep40950 28098228 PMC5241872

[B36] Li Z. Zhang Z. Xu C. Zhao J. Liu H. Fan Z. (2014). Bacteria and methanogens differ along the gastrointestinal tract of Chinese roe deer (*Capreolus pygargus*). *PLOS ONE* 9:e114513. 10.1371/journal.pone.0114513 25490208 PMC4260832

[B37] Li Z. P. Liu H. L. Li G. Y. Bao K. Wang K. Y. Xu C. (2013). Molecular diversity of rumen bacterial communities from tannin-rich and fiber-rich forage fed domestic Sika deer (*Cervus nippon*) in China. *BMC Microbiol.* 13:151. 10.1186/1471-2180-13-151 23834656 PMC3723558

[B38] Liang G. Malmuthuge N. Bao H. Stothard P. Griebel P. J. Guan L. L. (2016). Transcriptome analysis reveals regional and temporal differences in mucosal immune system development in the small intestine of neonatal calves. *BMC Genomics* 17:602. 10.1186/s12864-016-2957-y 27515123 PMC4981982

[B39] Malmuthuge N. Griebel P. J. Guan L. L. (2014). Taxonomic identification of commensal bacteria associated with the mucosa and digesta throughout the gastrointestinal tracts of preweaned calves. *Appl. Environ. Microbiol.* 80 2021–2028. 10.1128/aem.03864-13 24441166 PMC3957634

[B40] Malmuthuge N. Griebel P. J. Guan L. L. (2015). The gut microbiome and its potential role in the development and function of newborn calf gastrointestinal tract. *Front. Vet. Sci.* 2:36 10.3389/fvets.2015.00036 PMC4672224 26664965

[B41] Malmuthuge N. Guan L. L. (2017a). Understanding host-microbial interactions in rumen: searching the best opportunity for microbiota manipulation. *J. Anim. Sci. Biotechnol.* 8:8. 10.1186/s40104-016-0135-3 28116074 PMC5244612

[B42] Malmuthuge N. Guan L. L. (2017b). Understanding the gut microbiome of dairy calves: opportunities to improve early-life gut health. *J. Dairy Sci.* 100 5996–6005. 10.3168/jds.2016-12239 28501408

[B43] McCormack U. M. Curião T. Buzoianu S. G. Prieto M. L. Ryan T. Varley P. (2017). Exploring a possible link between the intestinal microbiota and feed efficiency in pigs. *Appl. Environ. Microbiol.* 83:e00380-17. 10.1128/AEM.00380-17 28526795 PMC5514681

[B44] Mueller N. T. Bakacs E. Combellick J. Grigoryan Z. Dominguez-Bello M. G. (2015). The infant microbiome development: mom matters. *Trends Mol. Med.* 21 109–117. 10.1016/j.molmed.2014.12.002 25578246 PMC4464665

[B45] Myer P. R. Wells J. E. Smith T. P. L. Kuehn L. A. Freetly H. C. (2016). Microbial community profiles of the jejunum from steers differing in feed efficiency. *J. Anim. Sci.* 94 327–338. 10.2527/jas.2015-9839 26812338

[B46] Oikonomou G. Teixeira A. G. V. Foditsch C. Bicalho M. L. Machado V. S. Bicalho R. C. (2013). Fecal microbial diversity in pre-weaned dairy calves as described by pyrosequencing of metagenomic 16S rDNA. Associations of *Faecalibacterium* species with health and growth. *PLOS ONE* 8:e63157. 10.1371/journal.pone.0063157 23646192 PMC3639981

[B47] Piccolo B. D. Mercer K. E. Bhattacharyya S. Bowlin A. K. Saraf M. K. Pack L. (2017). Early postnatal diets affect the bioregional small intestine microbiome and ileal metabolome in neonatal pigs. *J. Nutr.* 147 1499–1509. 10.3945/jn.117.252767 28659406

[B48] Presley L. L. Wei B. Braun J. Borneman J. (2010). Bacteria associated with immunoregulatory cells in mice. *Appl. Environ. Microbiol.* 76 936–941. 10.1128/aem.01561-09 20008175 PMC2813032

[B49] Price M. N. Dehal P. S. Arkin A. P. (2009). FastTree: computing large minimum evolution trees with profiles instead of a distance matrix. *Mol. Biol. Evol.* 26 1641–1650. 10.1093/molbev/msp077 19377059 PMC2693737

[B50] Quast C. Pruesse E. Yilmaz P. Gerken J. Schweer T. Yarza P. (2013). The SILVA ribosomal RNA gene database project: improved data processing and web-based tools. *Nucleic Acids Res.* 41 D590–D596. 10.1093/nar/gks1219 23193283 PMC3531112

[B51] Rey M. Enjalbert F. Combes S. Cauquil L. Bouchez O. Monteils V. (2014). Establishment of ruminal bacterial community in dairy calves from birth to weaning is sequential. *J. Appl. Microbiol.* 116 245–257. 10.1111/jam.12405 24279326

[B52] Rey M. Enjalbert F. Monteils V. (2012). Establishment of ruminal enzyme activities and fermentation capacity in dairy calves from birth through weaning. *J. Dairy Sci.* 95 1500–1512. 10.3168/jds.2011-4902 22365231

[B53] Ruth M. R. Field C. J. (2013). The immune modifying effects of amino acids on gut-associated lymphoid tissue. *J. Anim. Sci. Biotechnol.* 4:27. 10.1186/2049-1891-4-27 23899038 PMC3750756

[B54] Sandberg F. B. Emmans G. C. Kyriazakis I. (2007). The effects of pathogen challenges on the performance of naïve and immune animals: the problem of prediction. *Animal* 1 67–86. 10.1017/S175173110765784X 22444211

[B55] Santos T. M. A. Gilbert R. O. Bicalho R. C. (2011). Metagenomic analysis of the uterine bacterial microbiota in healthy and metritic postpartum dairy cows. *J. Dairy Sci.* 94 291–302. 10.3168/jds.2010-3668 21183039

[B56] Smedman A. E. M. Gustafsson I.-B. Berglund L. G. T. Vessby B. O. H. (1999). Pentadecanoic acid in serum as a marker for intake of milk fat: relations between intake of milk fat and metabolic risk factors. *Am. J. Clin. Nutr.* 69 22–29. 9925119 10.1093/ajcn/69.1.22

[B57] Stephens W. Z. Burns A. R. Stagaman K. Wong S. Rawls J. F. Guillemin K. (2016). The composition of the zebrafish intestinal microbial community varies across development. *ISME J.* 10 644–654. 10.1038/ismej.2015.140 26339860 PMC4817687

[B58] Uyeno Y. Sekiguchi Y. Kamagata Y. (2010). rRNA-based analysis to monitor succession of faecal bacterial communities in Holstein calves. *Lett. Appl. Microbiol.* 51 570–577. 10.1111/j.1472-765X.2010.02937.x 20849397

[B59] Wang Q. Garrity G. M. Tiedje J. M. Cole J. R. (2007). Naïve Bayesian classifier for rapid assignment of rRNA sequences into the new bacterial taxonomy. *Appl. Environ. Microbiol.* 73 5261–5267. 10.1128/aem.00062-07 17586664 PMC1950982

[B60] Weimer P. J. (2015). Redundancy, resilience, and host specificity of the ruminal microbiota: implications for engineering improved ruminal fermentations. *Front. Microbiol.* 6:296. 10.3389/fmicb.2015.00296 25914693 PMC4392294

[B61] Weimer P. J. Stevenson D. M. Mantovani H. C. Man S. L. C. (2010). Host specificity of the ruminal bacterial community in the dairy cow following near-total exchange of ruminal contents1. *J. Dairy Sci.* 93 5902–5912. 10.3168/jds.2010-3500 21094763

[B62] Wlodarska M. Willing B. P. Bravo D. M. Finlay B. B. (2015). Phytonutrient diet supplementation promotes beneficial Clostridia species and intestinal mucus secretion resulting in protection against enteric infection. *Sci. Rep.* 5:9253. 10.1038/srep09253 25787310 PMC4365398

[B63] Wu G. D. Compher C. Chen E. Z. Smith S. A. Shah R. D. Bittinger K. (2016). Comparative metabolomics in vegans and omnivores reveal constraints on diet-dependent gut microbiota metabolite production. *Gut* 65 63–72. 10.1136/gutjnl-2014-308209 25431456 PMC4583329

[B64] Wu S. Baldwin R. L. Li W. Li C. Connor E. E. Li R. W. (2012). The bacterial community composition of the bovine rumen detected using pyrosequencing of 16S rRNA genes. *Metagenomics* 1:11 10.4303/mg/235571

[B65] Xia J. Sinelnikov I. V. Han B. Wishart D. S. (2015). MetaboAnalyst 3.0–making metabolomics more meaningful. *Nucleic Acids Res.* 43 W251–W257. 10.1093/nar/gkv380 25897128 PMC4489235

[B66] Yanez-Ruiz D. R. Abecia L. Newbold C. J. (2015). Manipulating rumen microbiome and fermentation through interventions during early life: a review. *Front. Microbiol.* 6:1133. 10.3389/fmicb.2015.01133 26528276 PMC4604304

[B67] Yeruva L. Spencer N. E. Saraf M. K. Hennings L. Bowlin A. K. Cleves M. A. (2016). Formula diet alters small intestine morphology, microbial abundance and reduces VE-cadherin and IL-10 expression in neonatal porcine model. *BMC Gastroenterol.* 16:40. 10.1186/s12876-016-0456-x 27005303 PMC4804644

