# Supplementary figures and images for: The Development of Microbiota and Metabolome in Small Intestine of Sika Deer (Cervus nippon) from Birth to Weaning
Source: Front Microbiol. 2018 Jan 23;9:4. doi: 10.3389/fmicb.2018.00004 (PMC5787063; doi:10.3389/fmicb.2018.00004)

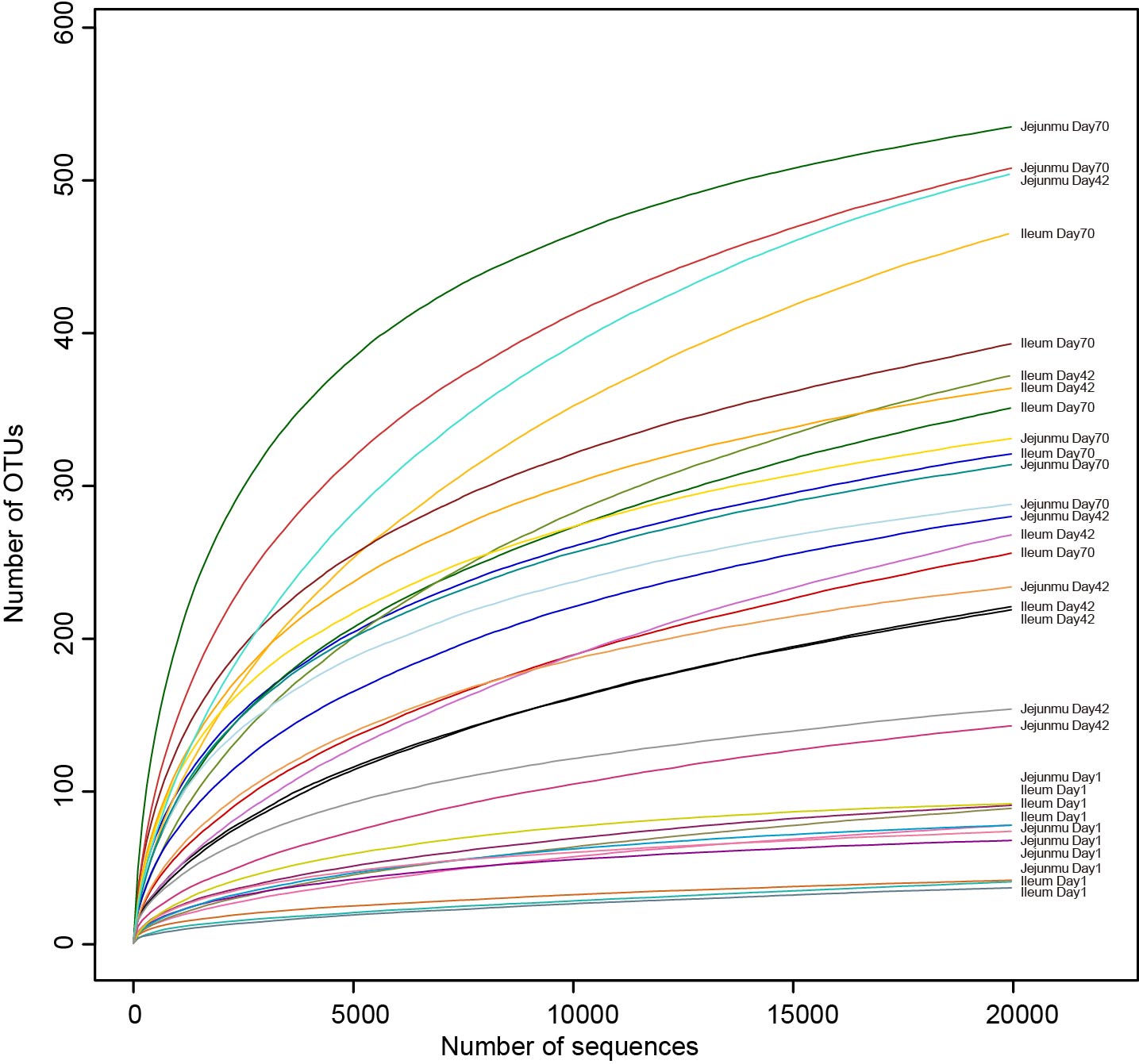

Supplement: FIGURE S1 — Rarefaction curves of the jejunum and ileum microbial community based on 16S rRNA gene sequencing based on a 97% sequence similarity. OTU, operational taxonomic unit. [file Image_1.JPEG]

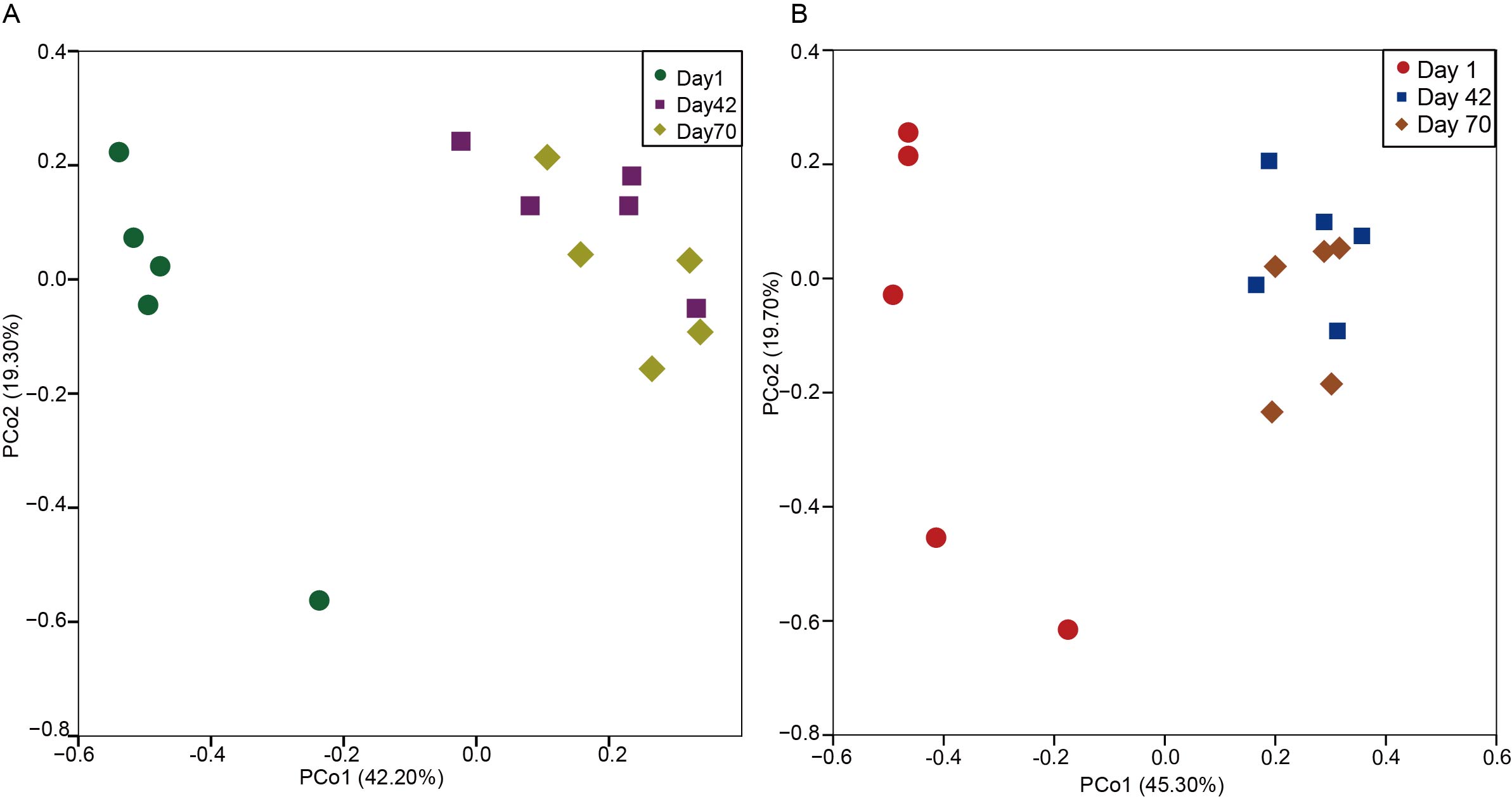

Supplement: FIGURE S2 — Principal coordinate analysis (PCoA) of jejunum (A) and ileum (B) microbiota based on Bray–Curtis distance. [file Image_2.JPEG]

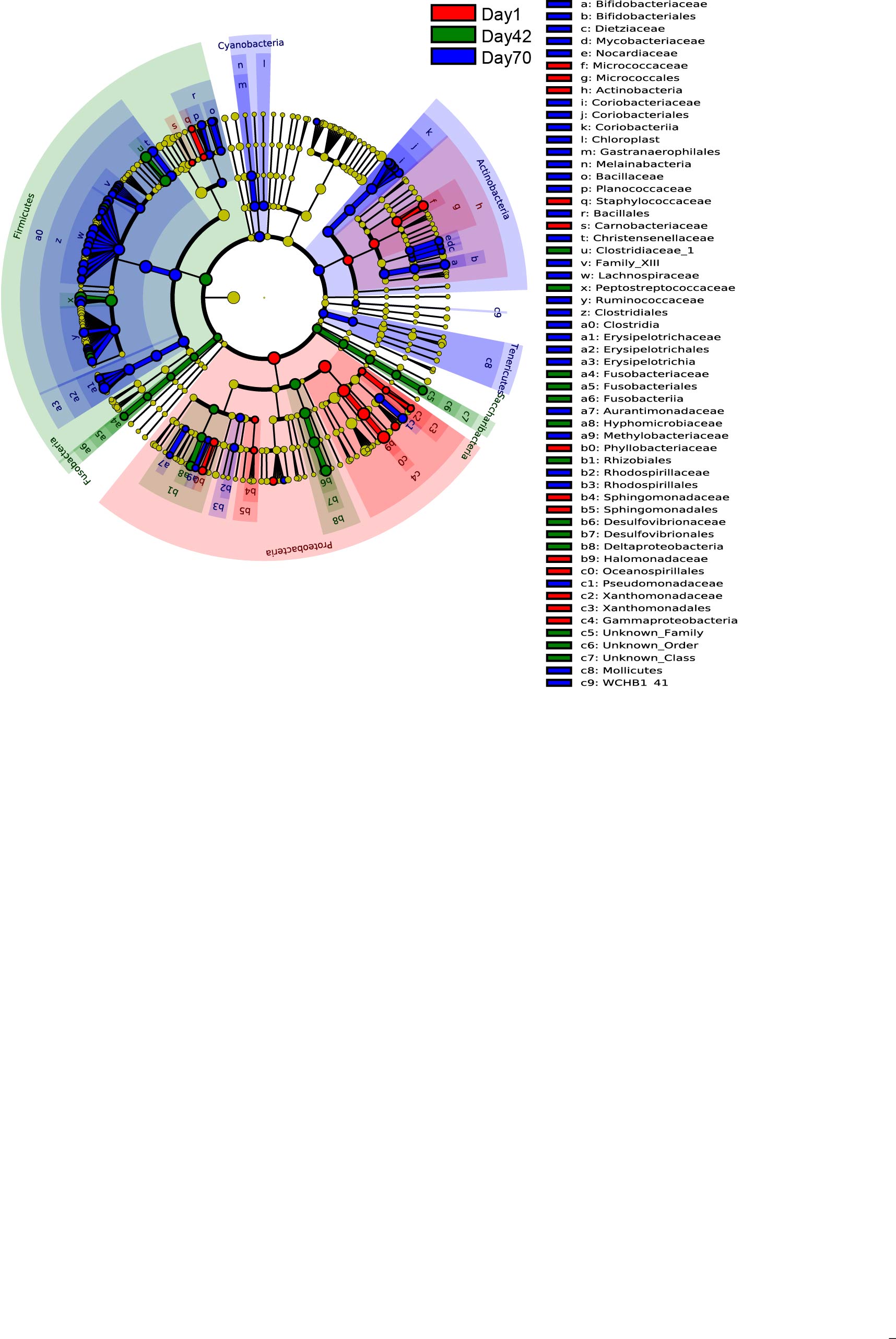

Supplement: FIGURE S3 — Taxonomic representation showing the statistically and biologically difference in jejunum across three time points. Differences are represented by the color of the most abundant class (red indicating the samples at day 1, green indicating the samples at day 42, and blue indicating the samples at day 70). The diameter of each circle’s diameter is proportional to the taxa abundance. [file Image_3.JPEG]

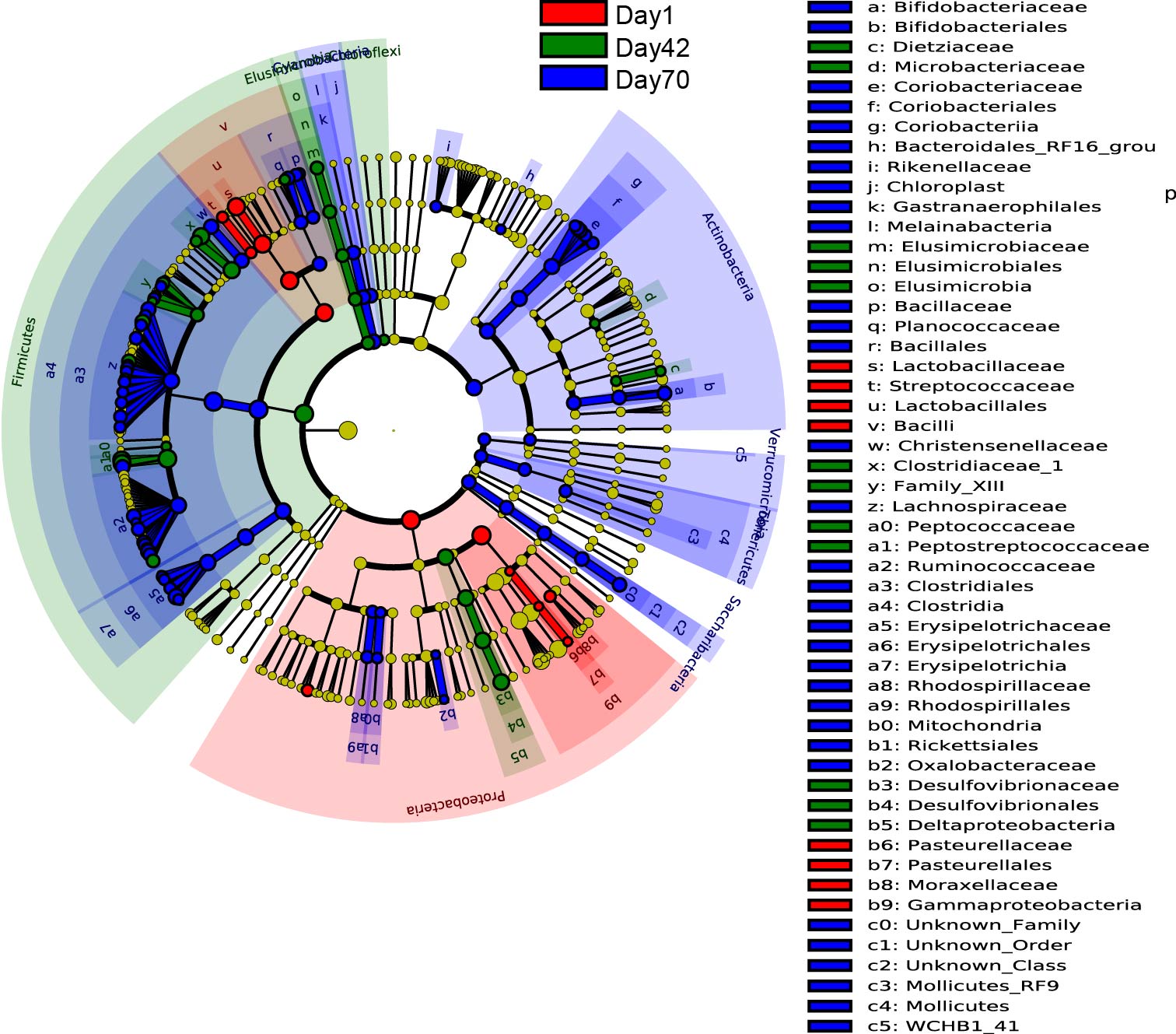

Supplement: FIGURE S4 — Taxonomic representation showing the statistically and biologically difference in ileum across three time points. Differences are represented by the color of the most abundant class (red indicating the samples at day 1, green indicating the samples at day 42, and blue indicating the samples at day 70). The diameter of each circle’s diameter is proportional to the taxa abundance. [file Image_4.JPEG]

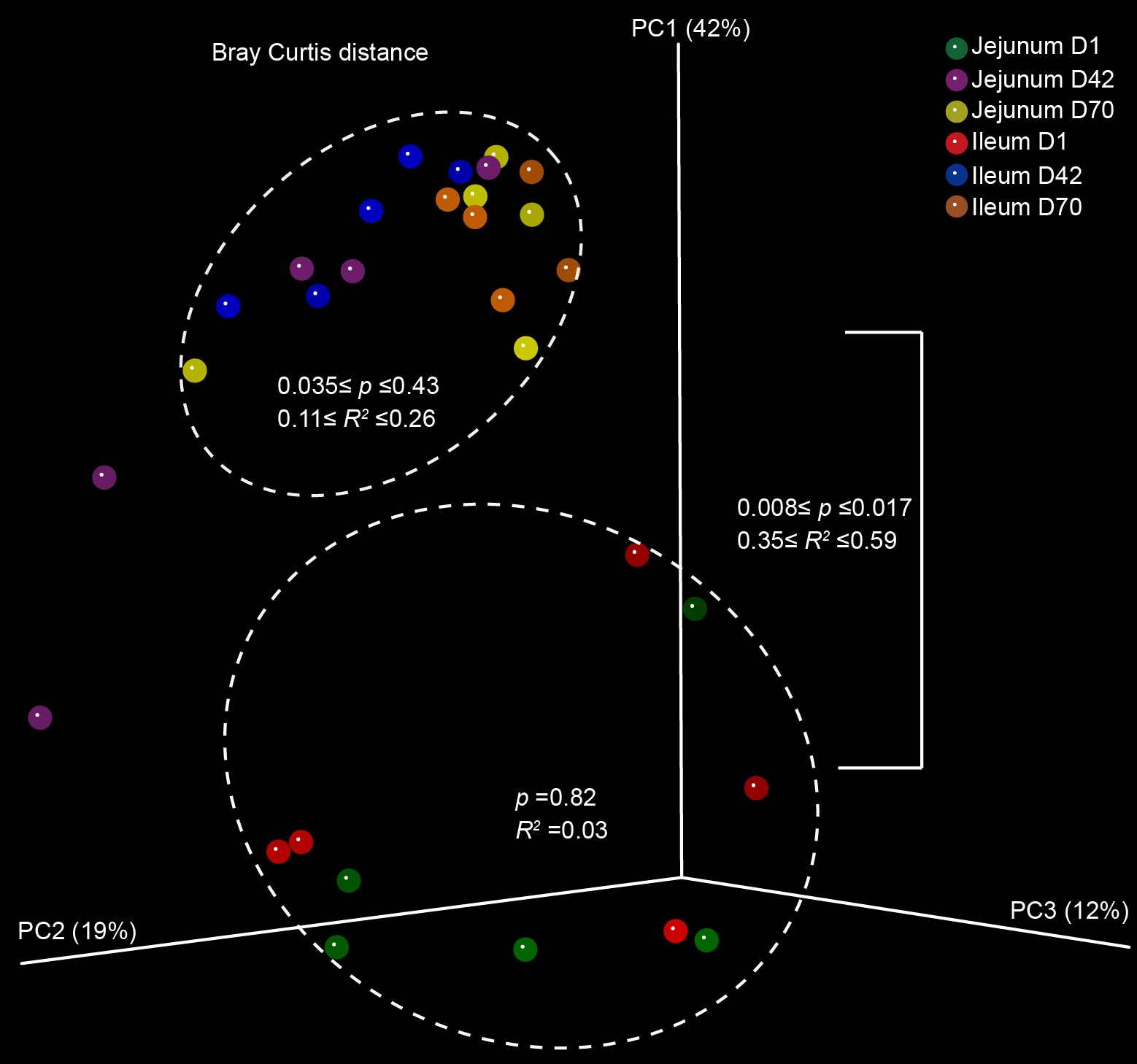

Supplement: FIGURE S5 — Comparison of the microbiota in jejunum and ileum across three time points using PCoA. R2- and p-values are the results of the multivariate ANOVA analysis based on the Bray–Curtis distance. D, day. [file Image_5.JPEG]
